# Construction of an index system of core competence assessment for infectious disease specialist nurse in China: a Delphi study

**DOI:** 10.1186/s12879-021-06402-2

**Published:** 2021-08-10

**Authors:** Chao Wu, Ping Wu, Pei Li, Feixia Cheng, Yanling Du, Shizhe He, Hongjuan Lang

**Affiliations:** 1 Air Force Medical University, Shaanxi, Xian 710032 China; 2 Tongji Hospital, Affiliated Hospital of Tongji Medical School of Huazhong University of Science and Technology, Hubei, Wuhan 430000 China; 3 Tangdu Hospital, the Second Affiliated Hospital of Air Force Military Medical University Shaanxi, Shaanxi, Xian 710032 China; 4 Naval University of Engineering, Hubei, Wuhan 430000 China

**Keywords:** Delphi method, Index system, Core competence, Infectious disease specialist nurse

## Abstract

**Aim and objective:**

This study was to establish an index system for the evaluation of Chinese infectious disease specialist nurses’ core competence.

**Background:**

The index system for the evaluation of infectious disease specialist nurses’ core competence has not been established.

**Design:**

A two-round Delphi survey was conducted to seek opinions from experts about the index system for the evaluation of infectious disease specialist nurses’ core competence.

**Methods:**

The study adopted several research methods, including literature retrieval, theoretical analysis and qualitative research. Based on the above method, the draft of core competence evaluation index system of infectious disease specialist nurses was constructed. A Delphi survey was used for the study of 30 infectious disease experts from 8 provinces and cities around China. A modified recommendation for the Conducting and Reporting of Delphi studies (CREDES) was also used to guide this study. A STROBE checklist was used.

**Results:**

The Core Competence Evaluation Index System of Infectious Disease Nurses is composed of 6 primary indicators, namely, Nursing Abilities for Infectious Diseases, Infection Prevention and Control Abilities, Responsiveness to Infectious Diseases, Professional Development Abilities, Communication and Management Abilities, and Professionalism and Humanistic Accomplishment, 16 secondary indicators and 47 tertiary indicators. The authority coefficient, judgment coefficient and familiarity degree of Delphi experts were 0.923, 0.933 and 0.913 respectively.

**Conclusions:**

The evaluation index system of core competence of diseases specialist nurses is scientific and reliable. It can be reference for future training and assessment of Chinese infectious disease specialist nurses.

**Relevance to clinical practice:**

Infectious disease specialist nurses are the main force for infectious disease nursing. Their core competence is related to the quality of infectious disease nursing and treatment. The core competence of the nurses is important for identification of training strategies and can be regarded as reference for nurse assessment and promotion. The construction of the index system is based on the consensus of infectious disease experts, which is not only helpful to standardize the training strategies and selection standards of infectious disease specialist nurses in the future, but also meet the society’s needs in clinical infectious disease nursing.

## Introduction

Infectious diseases have existed in human life for a very long time. In recent years, under the background of globalization and economic and cultural exchanges, public health emergencies of infectious diseases occur from time to time [1]. Especially after the outbreak of Coronavirus Disease 2019 (COVID-19), the pandemic spreads rapidly all over the world in a short period of time, which poses great threats to the health and property safety of all human beings [2, 3]. The importance of medical treatment and nursing of infectious diseases has become increasingly prominent [4]. The medical staff in infectious diseases department play a critical role in containing the spread of infectious diseases and treating infected patients [5].

The nurse specialist is a nurse prepared beyond the level of a generalist nurse and authorized to practice as a specialist with advanced expertise in the specific areas [6]. They are the leading figures in specialist nursing team, and their core competence level concerns the quality of the nursing and the development of the nursing career [2, 7]. Infectious disease specialist nurses are registered nurses who are able to undertake and guide clinical infectious diseases nursing work since they have undergone systematic and standardized training with professional knowledge and specialist practical skills of infectious diseases, communication and management abilities and teaching and coaching skills.

Core competence in the field of nursing refers to the knowledge, skills and attitude that a nurse must possess in clinical nursing work [8, 9]. In respond to the growing complexity and rapid change of infectious diseases, the core competence of infectious disease specialist nurses should also be constantly enriched and improved. A competent infectious disease specialist nurse should not only acquire the professional knowledge and skills so as to provide systematic, safe and ethical holistic care for infectious disease patients, but also have the abilities of safety protection and respond to infectious disease emergencies.

## Background

At present, there are some researches on the core competencies of diabetes specialist nurses [10], gastroenterology specialist nurses [11] and wound care specialist nurses [12] et al. However, there is no research on the core competence of infectious disease specialist nurses. Nowadays, the infectious disease profile in China has been shifting with rapid developments in society [13, 14]. China is a country with a large population where infectious diseases are easy to spread, especially the hepatopathy, approximately 300 million people suffer from it [15]. Therefore, infection prevention and control are of vital importance (M [16–18].). And infectious disease nursing plays a crucial part in the treatment to infectious disease patients.

Infectious disease specialist nurses are the main force for infectious disease nursing. Their core competence is related to the quality of infectious disease nursing and treatment. However, the clinical evaluation of the competence of nurses associated with infectious diseases is mostly based on subjective assessment which lacks systematic and objective standards (H [19–21].). It is not conducive to the standardized development of infectious disease specialist nurses.

In consideration of the complexity and uncertainty of infectious diseases and the increase of the incidence of transfusion transmitted diseases, the incidence of occupational exposure and injury among nursing personnel maintained an upward trend. This put forward higher requirements for the abilities of nurses in infectious diseases department. The aim of this study was to establish an index system for evaluation of infectious disease specialist nurse core competence.

## Methods

We use the method of qualitative research to establish the draft of the index system. A Delphi study was conducted to explore experts’ altitude to the index system for the core competence of infectious disease specialist nurses. A modified recommendation for the Conducting and Reporting of Delphi studies (CREDES) was used to guide the study [22, 23].

### Design

Delphi survey is a feedback anonymous inquiry method, during the process the experts do not know each other and can’t exchange opinions. Multiple rounds of consultation are conducted by questionnaires which contain the questions to be asked to obtain the opinions of experts until a consensus is reached [24]. This way of independent consultation can ensure that the experts won’t discuss or exchange views [25]. Therefore, it is regarded as an effective method to set goals, items, etc. Besides, we set up a research group which is composed of one professor, one associate professor, two lecturers, one supervisor nurse and one postgraduate. They are responsible for literature review, theoretical analysis, selection inquiry experts, compilation and distribution of inquiry questionnaires, sorting out and analyzing suggestions and data.

### Inclusion criteria

The inclusion criteria of consultation experts were as follows: (a) they should be engaged in clinical nursing or medical work of infectious diseases, and their working years in this field are no less than 15 years; (b) they should obtain the intermedium or advanced level certificate; (c) they should acquire a bachelor degree or above; (d) they should voluntarily participate in the investigation, and promise to participate in two rounds of consultation.

### Construction an evaluation index system

We consulted the literature about specialist nurses, infectious diseases, core competence and other related aspects [26–28] and the core competence theory as well [29]. On the basis of the above literature review, after discussion, our research group carried out qualitative face-to-face interviews with the infectious disease experts, specialist nurses and patients with infectious diseases. The inclusion criteria were the same as mentioned above. We conducted profound interviews based on the Grounded theory, according to which, the principle of sample size is information saturation [30, 31]. We carried out interviews with 21 subjects composed of 7 infectious disease experts, 10 infectious disease specialist nurses and 4 patients with infectious diseases from 5 hospitals in Shaanxi Province from June 2020 to August 2020. During the interviews, a lot of questions were included, such as: According to your experience, in the practices of clinical work, what kinds of work should the infectious disease specialist nurses undertake? What kinds of qualities should the infectious disease specialist nurses possess? During the period of hospitalization, what kinds of nursing services do you expect to be provided by the nurses? et al. With the consent of the interviewees, the researchers made records of the content of the interview. The researchers then transformed the recorded content into words one by one within 48 h, and used the Colaizzi’s 7-step phenomenological analysis method to sort out the interview data. Through Colaizzi’s method, we refined the content of the interview and finally set up 6 primary indicators (namely, Nursing Abilities for Infectious Diseases, Infection Prevention and Control Abilities, Responsiveness to Infectious Diseases, Professional Development Abilities, Communication and Management Abilities, and Professionalism and Humanistic Accomplishment), 17 secondary indicators and 48 tertiary indicators.

### The first draft of the expert consultation questionnaire

The questionnaire was composed of three parts: (a) general information of the experts: age, working years, education background, professional title, etc.; (b) evaluation index system of core competence of infectious disease specialist nurses expert consultation form: the importance of the index was evaluated by way of Likert 5-level scoring method, 5 = very important, 4 = important, 3 = general, 2 = unimportant, 1 = completely unimportant, and the column for suggestions was provided; (c) expert familiarity with the content of the survey and index judgment.

### Delphi consulting and feedback cycle

Firstly, after introducing the subject to experts and obtaining informed consent, we sent the questionnaire to experts in the field of infectious diseases by email. Experts scored and modified the index items, and then returned their feedback by email. The Delphi method was adopted to ensure the independence of opinions. We sorted out and summarized the experts’ opinions and redesigned the questionnaire for the second round. The index inclusion criterion: Mean value assignment > 4.5; coefficient of variation< 3. If the importance score was between 3.5 and 4.5, the research team needed to discuss and decide whether the indicator items needed to be retained or deleted [32]. In the second round of Delphi expert consultation, the opinions of all experts turned out to be the same, the evaluation index system was confirmed.

### Ethical consideration

Our research was approved by the ethics committee and was conducted under ethical guidelines described in the Helsinki Declaration (World Medical Association [33]). The purpose was elaborated to the experts before the investigation and verbal consent had been obtained from the experts before the survey. During the investigation, participants could terminate and withdraw from the investigation at any time.

### Quality control

In order to make sure that the research results were representative and reliable, we had proposed some basic requirements in selecting appraisal experts. As a result, 30 infectious disease experts from 8 provinces and cities including Shaanxi, Chongqing, Beijing, Zhejiang, Yunnan, Shandong, Hubei and Shanxi were selected. The research group collated and summarized the experts’ opinions. In the process of data analysis, we made use of Kendall coordination coefficient and chi square value to test the significance of the expert’s opinions, which assured the reliability of the results.

### Statistical analysis

SPSS 24.0 statistical software was applied in the process of data analysis. Data measurement and data calculation were expressed in the form of mean ± standard deviation and frequency, percentage respectively. The enthusiasm of the experts was also expressed in the form of questionnaire recovery rate. The expert authority coefficient (Cr) was the average value of expert familiarity with the indicators (Cs) and judgment criteria for the indicators [34]. The coordination degree of expert opinions was presented by Kendall harmony coefficient.

## Results

### Basic information of the experts

Among the experts participating in the consultation, 23 (76.67%) were infectious disease nursing experts, and 7 (23.33%) were infectious disease medical experts. Their age ranged from 36 to 56 years old, with an average age of 46.27 (*SD* 5.99). Their working years varied from 15 to 38 years, with an average of 25.27 (*SD* 5.92) years. Their educational background was different, 8 (26.67%) with bachelor’s degree, 16 (53.33%) with master’s degree and 6 (20%) with doctor’s degree (Table 1).

**Table 1:** Demographic information of experts

Project	Frequency (N)	Proportion (%)
Age (years)		
<40	4	13.33
40 ~ 50	17	56.67
>50	9	30.00
Highest degree		
Undergraduate	8	26.67
Master’s	16	53.33
Doctorate	6	20.00
Work experience in infectious disease (years)		
<20	7	23.33
20 ~ 30	18	60.00
>30	5	16.67
Title		
Junior level	5	16.67
Intermediate level	17	56.67
Advanced level	8	26.66
Research field		
Nursing of infectious disease	23	76.67
Treatment of infectious diseases	7	23.33

### Experts’ enthusiasm

The enthusiasm of the experts was assessed on the basis of the recovery of the questionnaires. In the first round, 32 questionnaires were distributed and 30 effective questionnaires were returned, with a recovery rate of 93.75%; in the second round, 30 questionnaires were distributed and all of them were returned, with a recovery rate of 100% (Table 2).

**Table 2:** Recovery of the questionnaire and suggestions offered

	First round	Second round
Questionnaire recovery		
Number of questionnaires distributed	32	30
Number of recycled questionnaires	30	30
Rate of recovery (%)	93.75	100
Effective questionnaire	30	30
Effective proportion	100	100
Proposed ratio		
Number of experts	19	2
Constituent ratio (%)	63.33	6.67

### Expert authority coefficient and opinion coordination degree

The judgment coefficient, familiarity coefficient and authority coefficient are 0.933, 0.913 and 0.923 respectively, which meet the standard of expert consultation authority coefficient > 0.7. In the first round of expert consultation, the Kendall’s concordance coefficients of the first, second and third level indicators were 0.156, 0.262 and 0.318 respectively. While in the second round of expert consultation, the Kendall’s concordance coefficients of the first, second and third level indicators were 0.177, 0.236 and 0.324 respectively. The results of the Kendall’s concordance coefficients were on medium level and the Kendall’s test had statistical significance (all *p* < 0.01) (Table 3).

**Table 3:** The result of expert opinions’ coordination degree

Hierarchical level	Index(n)	CV/Kendall’s *W*	*X*^2^	*P*
First-level	6	0.156	23.356	0.000
Second-level	17	0.262	125.990	0.000
Third-level	48	0.318	448.490	0.000
First-level	6	0.177	26.579	0.000
Second-level	16	0.236	106.262	0.000
Third-level	47	0.324	447.329	0.000

### The index system of core competence assessment for infectious disease specialist nurses

In this study, we conducted two rounds of consultation and made use of the Delphi method. In round 1, the research team modified 12 indexes, deleted 3 indexes, merged 3 indexes, added 2 indexes, adjusted 2 indexes based on the exclusion criterion and experts’ opinions. In round 2, the research team modified 2 indexes. Finally, the index system of core competence assessment for infectious disease specialist nurses was established and it included 6 first level indexes, 16 s level indexes and 47 third level indexes. The first level indicators referred to Nursing Abilities for Infectious Diseases, Infection Prevention and Control Abilities, Responsiveness to Infectious Diseases, Professional Development Abilities, Communication and Management Abilities, and Professionalism and Humanistic Accomplishment. Index weight was referred to the relative importance of a certain index in the overall evaluation system. And the Delphi method was applied to determine the index weight. The weight index was determined by dividing the sum of the scores of each item of the index into the score that represented the importance of the corresponding index according to the experts’ opinion. In the terms of weight, Infection Prevention and Control Abilities showed the highest value (0.172), followed by Nursing Abilities for infectious diseases (0.169), while Professionalism and Humanistic Accomplishment showed the smallest value (0.161). Among the secondary indicators, Safety Protection Abilities and Humanistic Care showed the highest value (0.065), while Prediction and Reporting showed the smallest value (0.058) (Table 4).

**Table 4:** Core competence evaluation index system of infectious disease specialist nurses

Index level 1st, 2nd and 3rd	Significance grade	Variable coefficient	Weighting targets
1. Nursing abilities for infectious diseases	4.90 ± 0.31	0.062	0.169
1.1 Theoretical knowledge	4.87 ± 0.35	0.071	0.063
1.1.1 Grasp the pathogenesis of common infectious diseases and the relevant knowledge of diseases including the historical epidemiology, the main nursing points and health education	4.67 ± 0.55	0.117	0.021
1.1.2 Be familiar with the basics such as the dosage regimen, administration route, side effects and matters needing attention of drugs which are commonly taken by infectious diseases patients	4.50 ± 0.78	0.173	0.020
1.2 Operational skills	4.83 ± 0.38	0.078	0.063
1.2.1 Be able to collect different samples such as blood and nasopharyngeal swab from infectious diseases patients	4.87 ± 0.35	0.071	0.022
1.2.2 Be able to deal with the symptoms and signs of common diseases such as fever, erythra, diarrhea, twitching and seizure	4.87 ± 0.35	0.071	0.022
1.2.3 Be familiar with the common diagnosis and treatment in infectious disease departments such as compression hemostasis for Sengstaken-Blakemore tube, lactulose enema, and traumatic artetial blood pressure supervision paracentesis.	4.70 ± 0.54	0.114	0.021
1.2.4 Master the emergence care skills for critical infectious diseases patients	4.83 ± 0.38	0.078	0.022
1.3 Critical thinking capabilities	4.90 ± 0.31	0.062	0.064
1.3.1 Be able to assess and observe the state of the infectious diseases patients	4.87 ± 0.35	0.071	0.022
1.3.2 Be able to predict and figure out the risk of occurrence of the potential complications (such as hepatic encephalopathy, esophagogastric variceal bleeding and suffocation)	4.80 ± 0.41	0.085	0.022
1.3.3 Be able to draw up nursing plans in accordance with the different state of the different infectious diseases patients	4.87 ± 0.35	0.071	0.022
2. Infection prevention and control abilities	5.00 ± 0.00	0.000	0.172
2.1 Safety protection capabilities	5.00 ± 0.00	0.000	0.065
2.1.1 Be familiar with the law on prevention and control of infectious diseases of the People’s Republic of China and the guidance to protective techniques for health care workers	4.27 ± 0.74	0.173	0.019
2.1.2 Be aware of the protective requirements for different kinds of infectious diseases	4.90 ± 0.31	0.062	0.022
2.1.3 Master the tactics of standard and extra precautions	5.00 ± 0.00	0.000	0.022
2.1.4 Master the processes and methods of putting on the protective articles	5.00 ± 0.00	0.000	0.022
2.1.5 Master the skills and processes coping with professional exposure risks such as skin mucous membrane and sharp instrument injury	4.97 ± 0.18	0.037	0.022
2.2 Disinfection and quarantine skills	4.97 ± 0.18	0.037	0.065
2.2.1 Be familiar with common physical and chemical disinfection	4.80 ± 0.41	0.085	0.022
2.2.2 Be able to correctly dispose of different medical wastes by infectious diseases patients (such as infectious diarrhea, AIDS, COVID-19, etc.)	4.70 ± 0.54	0.114	0.021
2.2.3 Master the disinfection methods of inpatient ward and instrument and equipment in infectious diseases department	4.77 ± 0.50	0.106	0.021
2.2.4 Master the common isolation techniques and methods	4.87 ± 0.35	0.071	0.022
2.2.5 Be aware of the requirements for different isolation techniques (isolation due to airborne transmission, contact transmission and droplet transmission)	4.87 ± 0.35	0.071	0.022
3. Responsiveness to infectious diseases	4.80 ± 0.41	0.085	0.166
3.1 Emergency drills	4.67 ± 0.61	0.130	0.061
3.1.1 Be familiar with national emergency plan for medical and health rescue in response to public emergencies	4.27 ± 0.69	0.162	0.019
3.1.2 Take part in emergency drills for infectious diseases emergencies in regular terms	4.40 ± 0.72	0.165	0.020
3.2 Prediction and reporting	4.47 ± 0.78	0.174	0.058
3.2.1 Be able to predict and recognize the infectious diseases emergencies	4.27 ± 0.58	0.137	0.019
3.2.2 Master the reporting process of the infectious diseases emergencies	4.70 ± 0.47	0.099	0.021
3.3 Response capabilities	4.87 ± 0.35	0.071	0.063
3.3.1 Be familiar with the response process of the infectious diseases emergencies	4.60 ± 0.56	0.122	0.021
4. Professional development abilities	4.67 ± 0.55	0.117	0.161
4.1 Learning abilities	4.87 ± 0.35	0.071	0.063
4.1.1 Learn the cutting-edge dynamic knowledge on infectious diseases	4.80 ± 0.41	0.085	0.022
4.1.2 Participate in knowledge training and academic exchanges activities on infectious diseases	4.53 ± 0.51	0.112	0.020
4.2 Teaching abilities	4.50 ± 0.73	0.162	0.059
4.2.1 Be able to conduct clinical teaching	4.67 ± 0.53	0.113	0.021
4.2.2 Be able to conduct lectures	4.57 ± 0.63	0.137	0.021
4.2.3 Be able to train other nurses in face of emerging infectious diseases emergencies	4.87 ± 0.35	0.071	0.022
4.3 Abilities in scientific researches and inventions	4.57 ± 0.68	0.149	0.059
4.3.1 Be able to select and design scientific researches	4.60 ± 0.62	0.135	0.021
4.3.2 Be able to search and retrieve literature documents by various ways and assess the quality of the literature	4.67 ± 0.55	0.117	0.021
4.3.3 Be able to collect, analyze and process the relevant data	4.60 ± 0.68	0.147	0.021
4.3.4 Be able to write papers	4.73 ± 0.45	0.095	0.021
4.3.5 Be able to improve and innovate on infectious disease nursing process and protective articles	4.87 ± 0.35	0.071	0.022
5. Communication and management abilities	4.80 ± 0.41	0.085	0.166
5.1 Communication skills	4.83 ± 0.38	0.078	0.063
5.1.1 Master the communication skills with infectious diseases patients and their families in consideration with the patients’ sensitivity, fear and other psychological characteristics	4.83 ± 0.38	0.078	0.022
5.1.2 Be able to communicate effectively with other team members so as to improve the treatment	4.80 ± 0.41	0.085	0.022
5.2 Coordination abilities	4.70 ± 0.60	0.127	0.061
5.2.1 Be able to cope with disputes between nursing workers and patients	4.67 ± 0.48	0.103	0.021
5.2.2 Be able to cooperate with other medical workers and have the capability of organization	4.70 ± 0.54	0.114	0.021
5.2.3 Be able to collaborate with other units and departments and effectively coordinate human and material resources, etc.	4.60 ± 0.62	0.135	0.021
5.3 Management abilities	4.87 ± 0.35	0.071	0.063
5.3.1 Be able to distribute, guide, supervise and manage the infectious diseases nurses	4.83 ± 0.38	0.078	0.022
5.3.2 Be able to manage materials such as drugs, consumable items, documents, instrument and equipment in infectious diseases department	4.50 ± 0.63	0.140	0.020
5.3.3 Be able to evaluate and improve the quality of infectious diseases nursing issues and interventions	4.83 ± 0.38	0.078	0.022
6. Professionalism and humanistic accomplishment	4.83 ± 0.38	0.078	0.167
6.1 Professionalism	4.93 ± 0.25	0.052	0.064
6.1.1 Be passionate about infectious diseases nursing and possess the sense of professional identity of nursing infectious diseases patients	4.90 ± 0.31	0.062	0.022
6.1.2 Be capable of adjusting oneself and governing the stress in infectious diseases nursing work	4.93 ± 0.25	0.052	0.022
6.2 Humanistic care	4.97 ± 0.18	0.037	0.065
6.2.1 Respect the patient and protect patients’ privacy and show no discrimination to the patients	4.90 ± 0.31	0.062	0.022
6.2.2 Be capable of providing psychological counseling and mental nursing	4.87 ± 0.35	0.071	0.022
6.2.3 Be capable of providing health education for the infectious diseases patients and the public	4.87 ± 0.35	0.071	0.022

*Note*: Weighting Targets = Significance Grade/ Sum of index grades of the same item

## Discussion

### Reliability of Delphi expert consultation results

In this study, the experts who participated in the Delphi consultation came from 8 different provinces and cities in China, which ensured that the results were objective and would not be affected by the region. The consulting experts had been engaged in the work relevant to infectious diseases for more than 15 years, so they had rich experience in clinical infectious diseases and were familiar with the research. The authority coefficient of this research was 0.923, which proved that the authority of the research was assured. The response rates of the two rounds of expert consultation were 93.75 and 100% respectively, indicating that the experts were enthusiastic in the research. The Kendall’s concordance coefficient of the two rounds of consultation were statistically significant, so it proved that the results of the evaluation index system of core competence of infectious disease specialist nurses were reliable.

### Importance of evaluation index system of core competence of infectious disease specialist nurses

Infectious disease specialist nurses are the backbone of infectious disease nursing. Their core competence is related to the work quality, service level and team development of infectious disease nursing. At present, there is no research on the core competence index system of infectious disease specialist nurses, so the definition of the core competence of infectious disease specialist nurses is not clear. In the context of the novel Coronavirus disease and the increasing number of new infectious diseases [35, 36], speeding up the training of infectious disease specialist nurses and clarifying their core competencies will help standardize the work of infectious disease nursing, improve the quality of nursing services, and prevent the spread of infectious diseases. Therefore, it is of great practical significance to construct the evaluation index system of core competence of infectious disease specialist nurses.

### Comprehensiveness of evaluation index system of core competence of infectious disease specialist nurses

#### Nursing abilities for infectious diseases

Infectious diseases are susceptible and transmissible, easy to spread and harmful [37, 38]. The management of infectious diseases is the basic responsibility of specialist nurses, and also one of the most important core competencies. In this study, the weight coefficient of infectious disease nursing ability was 0.169, ranking second in the first level indicators, only to the ability of infection prevention and control abilities, indicating that this ability is highly recognized by experts and very important. Infectious disease specialist nurses should be familiar with the knowledge of infectious diseases and commonly used drugs. They should be able to formulate corresponding nursing plans in response to different infectious disease patients and implement different nursing measures accordingly. At the same time, specialist nurses should also master first-aid nursing methods for patients with infectious diseases, make clear the requirements of specimen collection and the terminal treatment methods for patients with infectious diseases.

#### Infection prevention and control abilities

Infection prevention and control ability is the most important ability of infectious disease specialist nurses should possess, since it is an important guarantee for continuous work [39–41]. It is reported that the risk that infectious diseases of nurses in infectious diseases department got infected is much higher than that of ordinary clinical nurses [40]. The importance of infection prevention and control ability in this study ranked the first, with the weight coefficient of 0.172. The secondary indexes were safety protection abilities and disinfection and quarantine skills, with safety protection abilities as the largest weight. In the clinical infectious disease nursing work, nurses in the infectious disease department should put self-protection in the first place.

#### Responsiveness to infectious diseases

Responsiveness to infectious diseases refers to the ability of emergency rescue and nursing in public health emergencies. Public health emergencies of infectious diseases normally spread widely and rapidly, and do great harm [42]. Therefore, as a specialist nurse of infectious diseases, it is necessary to have the ability of emergency response and the ability to predict the emergency of infectious diseases. In their daily work, they need to keep in mind the reporting time limit of notifiable diseases, and be familiar with the reporting and drilling process and relevant regulations of public health emergencies of infectious diseases. In the clinical nursing work of New Coronavirus disease, infectious disease specialist nurses played an important role in the outbreak of disease [43].

#### Professional development abilities

Infectious disease nursing is a profession that should be kept pace with the times. Facing the continuous variation and evolution of the virus, the specialist nurse needs to have the ability of professional development. The ability ‘to train other nurses in case of emerging infectious diseases emergencies’ and ‘to improve and innovate on infectious disease nursing process and protective articles’ accounted for the largest weight, which were 0.022. In public health emergencies of infectious diseases, it is of great significance for infectious disease specialist nurses to help ordinary nurses to work quickly according to the standardized training process, so as to enhance and expand the nursing reserve force [44, 45] and innovation is an important driving force to promote clinical nursing work [46]. At the same time, as a specialist nurse of infectious diseases, it is also necessary to master the frontier dynamic knowledge, have the ability of further teaching and learning, and promote the professional development and the improvement of their own ability.

#### Communication and management abilities

In clinical nursing work, good communication and management skills are essential [47, 48]. Due to the particularity of patients with infectious diseases, they are more sensitive, so in the process of communication with patients with infectious diseases or their families, nurses should pay attention to the skills, contents and methods of communication [49]. In the second level indicators, the score of management abilities accounts the highest. Specialist nurses are different from ordinary nurses. They need not only master basic nursing skills, but also assume the role of a manager.

#### Professionalism and humanistic accomplishment

Since infectious disease nursing work is confronted with infectious diseases patients, the work is of a certain degree of risks. Therefore, infectious disease specialist nurses should have good professional humanistic accomplishment. They must recognize the occupation identity of infectious disease nursing work. Infectious disease nursing work is a high-risk occupation [50], so the nurses should have good self-adjusting ability to release the pressure generated in infectious disease nursing work. In the process of communication with infectious diseases patients, they should respect and treat every patient equally.

## Conclusion

Through two rounds of Delphi expert consultation, the core competence index system of infectious disease specialist nurses was established, which was featured with good authority, reliability and comprehensiveness. It can provide reference for the training and assessment of infectious disease specialist nurses in the future.

## Relevance to clinical practice

In the context of the global Novel Coronavirus pneumonia pandemic, the importance of infectious disease nursing is increasing. As the backbone of nursing work, specialist nurses play an important role in nursing work. To clarify their core competence requirements is helpful to specify the content for the development of infectious disease specialist nurses and provide reference for their training and assessment.

## Data Availability

The datasets generated and analyzed during the current study are not publicly available due to the protection of the privacy of consulting experts but are available from the corresponding author (906963251@qq.com) on reasonable request.
